# Projection to extract the perpendicular component (PEPC) method for extracting kinetics from time-resolved data

**DOI:** 10.1063/4.0000189

**Published:** 2023-06-27

**Authors:** H. Ki, J. Gu, Y. Cha, K. W. Lee, H. Ihee

**Affiliations:** 1 Department of Chemistry and KI for the BioCentury, Korea Advanced Institute of Science and Technology (KAIST), Daejeon 34141, Republic of Korea; 2 Center for Advanced Reaction Dynamics, Institute for Basic Science, Daejeon 34141, Republic of Korea

## Abstract

Time-resolved x-ray liquidography (TRXL) is a potent method for investigating the structural dynamics of chemical and biological reactions in the liquid phase. It has enabled the extraction of detailed structural aspects of various dynamic processes, the molecular structures of intermediates, and kinetics of reactions across a wide range of systems, from small molecules to proteins and nanoparticles. Proper data analysis is key to extracting the information of the kinetics and structural dynamics of the studied system encrypted in the TRXL data. In typical TRXL data, the signals from solute scattering, solvent scattering, and solute–solvent cross scattering are mixed in the *q*-space, and the solute kinetics and solvent hydrodynamics are mixed in the time domain, thus complicating the data analysis. Various methods developed so far generally require prior knowledge of the molecular structures of candidate species involved in the reaction. Because such information is often unavailable, a typical data analysis often involves tedious trial and error. To remedy this situation, we have developed a method named projection to extract the perpendicular component (PEPC), capable of removing the contribution of solvent kinetics from TRXL data. The resulting data then contain only the solute kinetics, and, thus, the solute kinetics can be easily determined. Once the solute kinetics is determined, the subsequent data analysis to extract the structural information can be performed with drastically improved convenience. The application of the PEPC method is demonstrated with TRXL data from the photochemistry of two molecular systems: [Au(CN)_2_^−^]_3_ in water and CHI_3_ in cyclohexane.

## I. INTRODUCTION

Time-resolved x-ray liquidography (TRXL), also known as time-resolved x-ray solution scattering, has been established as a useful method for studying the structural dynamics and kinetics of chemical and biological reactions in the liquid solution phase.^1–5^ Molecular structures of reaction intermediates and their kinetics have been investigated with TRXL at third generation synchrotrons for a variety of molecules spanning from diatomic molecules to proteins and nanoparticles.^6–28^ Thanks to the recent development of x-ray free-electron lasers (XFELs), the time resolution of TRXL has reached the sub-picosecond regime^29–38^ and has even allowed for tracking time-dependent positions of nuclear wavepackets.^34,36,39^ Although the development of x-ray sources and facilities has greatly improved the time resolution and signal-to-noise ratio (SNR) of TRXL data, it is the development of appropriate data analysis methods that has enabled the expansion of the depth and breadth of information extracted from TRXL data.

The key step in the analysis of TRXL data is to determine the kinetics and structures of reaction intermediates, and many useful analysis methodologies have been developed.^40–48^ In typical TRXL data, the signals from solute scattering, solvent scattering, and solute–solvent cross scattering are mixed in the *q*-space,^43^ and the solute kinetics and solvent hydrodynamics (or solvent kinetics) are mixed in the time domain. For this reason, the data analysis of TRXL data is not trivial. Various methods that have been developed so far generally require prior knowledge of the molecular structures of candidate species involved in the reaction.^7,10,13,43,49^ Since such information is often unavailable; a typical data analysis often involves tedious trial and error.

In this work, we report a method to circumvent this situation. In this method named projection to extract the perpendicular component (PEPC), we take advantage of the fact that the scattering profiles of the solvent term are well-known. We show that by subtracting a scaled portion of the known solvent term from the TRXL signals, the resulting signals, i.e., the PEPC-treated signals, exhibit no contribution from the solvent kinetics, although they retain the intact solute kinetics. The absence of solvent kinetics in the PEPC-treated signals allows the straightforward determination of the solute kinetics without any prior knowledge of the molecular structures of the participating solute species.

## II. RESULTS AND DISCUSSION

### A. TRXL data

Usually, experimental difference scattering curves, ΔS(*q*, *t*)_exp_, obtained by subtracting the scattering curve at a negative time delay (*t*_ref_) from the scattering curve at a positive time delay (*t*) of interest, as given in Eq. (1), are subject to data analysis,

$${\Delta S(q,\;t)}_{\text{exp}\;}={S(q,\;t)}_{\text{exp}\;}-{S(q,\;t_{\text{ref}})}_{\text{exp}}.$$ (1)

To extract the kinetics and structural dynamics information from ΔS(*q*, *t*)_exp_, the theoretical ΔS(*q*, *t*) and ΔS(*q*, *t*)_theory_ need to be calculated and compared with ΔS(*q*, *t*)_exp_. For the purpose of analysis, we categorize the total scattering signal into the following three terms based on whether the atom belongs to the solute or solvent molecules. Accordingly, ΔS(*q*, *t*)_theory_ comprises three components: (i) solute-only term [ΔS(*q*, *t*)_solute_], (ii) solute–solvent cross term [ΔS(*q*, *t*)_cage_], and (iii) solvent-only term [ΔS(*q*, *t*)_solvent_], as given in the following equation:^43^

$${\Delta S(q,\;t)}_{\text{theory}}={\Delta S(q,\;t)}_{\text{solute}}+{\Delta S(q,\;t)}_{\text{cage}}+{\Delta S(q,\;t)}_{\text{solvent}}.$$ (2)

The solute-only term contains information about the change in the molecular structure of solute molecules, whose concentrations change according to the solute kinetics. The cage term contains information about the change in the cage structure according to the solute kinetics. The solvent-only term contains the changes in the arrangement of solvent molecules in the bulk solvent, mainly due to the changes in temperature and density of the solvent, which are caused because the photon energy absorbed by the solute molecules is transferred to the solvent molecules. The solute-only term can be calculated using the Debye equation. For this calculation, the molecular structure of the solute molecules needs to be known. Often, the structure from density functional theory (DFT) calculations is used as the starting structure, and the structural parameters can be refined through a fitting process that employs those structural parameters as fitting parameters. The cage term can be calculated using pair distribution functions, g(*r*), from molecular dynamics (MD) simulations. The sum of the solute-only term and the solute–solvent cross term can be categorized into the solute-related term, as follows:

$${\Delta S(q,\;t)}_{\text{sol}–\text{rel}}={\Delta S(q,\;t)}_{\text{solute}}+{\Delta S(q,\;t)}_{\text{cage}},$$ (3)

then, ΔS(*q*, *t*)_theory_ can be written as the sum of ΔS(*q*, *t*)_sol-rel_ and ΔS(*q*, *t*)_solvent_

$${\Delta S(q,\;t)}_{\text{theory}}={\Delta S(q,\;t)}_{\text{sol}–\text{rel}}+{\Delta S(q,\;t)}_{\text{solvent}}.$$ (4)

The ΔS(*q*, *t*)_sol-rel_ term can be expressed as the sum of the contributions of relevant solute species, i.e., the sum of difference scattering curves of different solute species. More specifically, it can be written as follows:

$${\Delta S(q,\;t)}_{\text{sol}–\text{rel}}=1/R\times \sum_{k}\left(f_{k}(t)\times {\text{SADS}}_{k}(q)\right),$$ (5)

where *R* is the ratio of the number of solvent molecules with respect to that of solute molecules, *f_k_*(*t*) is the molar fraction of the *k*th solute species among the total solute molecules at time *t*, and SADS_*k*_(*q*) is the solute-related term per unit concentration of the *k*th solute species. SADS stands for the species-associated difference scattering curve. As shown in Eq. (5), in a typical TRXL analysis, the solute-related term is multiplied by the factor of 1/*R* so that the term is scaled to indicate the amplitude of the signal per a solvent molecule, not per a solute molecule. In principle, Eq. (5) is applicable to general structural changes, even including the case of continuous structural changes. For such a continuous structural change, the structure at each time point would have its own SADS_*k*_(*q*) with its corresponding *f_k_*(*t*) be a delta function. SADS_*k*_(*q*) can be expressed as follows:

$${\text{SADS}}_{k}(q)=\Delta S_{k}{\left(q\right)}_{\text{solute}}+{\Delta S}_{k}{\left(q\right)}_{\text{cage}},$$ (6)

$${\text{SADS}}_{k}(q)=S_{k}{\left(q\right)}_{\text{solute}}-S_{0}{\left(q\right)}_{\text{solute}}+S_{k}{\left(q\right)}_{\text{cage}}-S_{0}{\left(q\right)}_{\text{cage}},$$ (7)

where ΔS_*k*_(*q*)_solute_ and ΔS_*k*_(*q*)_cage_ are the difference solute-only term and the difference solute–solvent cross term of the *k*th solute species, respectively; S_*k*_(*q*)_solute_ and S_*k*_(*q*)_cage_ are the solute-only term and the solute–solvent cross term of the kth solute species, respectively; and S_0_(*q*)_solute_ and S_0_(*q*)_cage_ are the solute-only term and the solute–solvent cross term of reactants, respectively.

Actually, as expressed in Eqs. (3) and (6), the solvent cages surrounding the solute molecules, as well as the solute molecules, contribute to ΔS(*q*, *t*)_sol-rel_, thus to its component, SADS_*k*_(*q*). Here, for the simplicity of explanation, the term “solute species” is used as a term that includes both a solute species and its surrounding cage structure. We note that the kinetics of surrounding cage structure and solute molecules can be different, particularly at early time delays when non-equilibrium cage structures can be observed. For such a case, in Eq. (5), a large number of SADS_*k*_(*q*)'s are required to describe the continuous structural changes accompanying the non-equilibrium dynamics. Also the corresponding *f_k_*(*t*)'s would show more complex behavior on the *t*-axis to describe the kinetics of both solute molecules and surrounding cage structure, which are independent of each other.

The solvent-only term, ΔS(*q*, *t*)_solvent_, given in Eq. (8), consists of two basis differentials (∂S/∂T)_ρ_ and (∂S/∂ρ)_T_,^43,49^ which can be obtained in a separate solvent heating experiment either by exciting the dye molecules in a dye solution^50^ or by directly exciting the vibrational overtone of the solvent molecules in a neat solvent using near-infrared excitation,^51^

$${\Delta S(q,\;t)}_{\text{solvent}}=\Delta T(t)\times {\left(\partial S/\partial T\right)}_{\rho }+\Delta \rho (t)\times {\left(\partial S/\partial \rho \right)}_{T}.$$ (8)

In this equation, (∂S/∂T)_ρ_ is the change of the solvent scattering intensity in response to a temperature change at a constant density, (∂S/∂ρ)_T_ is the change of the solvent scattering intensity in response to a density change at a constant temperature, and ΔT(*t*) and Δρ(*t*) are the time-dependent changes in the temperature and density of the solvent at the time delay *t*, respectively.

A combination of Eqs. (4), (5), and (8) gives the following relationship:^43,49^

$${\Delta S(q,\;t)}_{\text{theory}}=1/R\times \sum_{k}\left(f_{k}(t)\times {\text{SADS}}_{k}(q)\right)+\Delta T(t)\times {\left(\partial S/\partial T\right)}_{\rho }+\Delta \rho (t)\times {\left(\partial S/\partial \rho \right)}_{T}.$$ (9)

The goal of data analysis is to determine (i) the kinetic framework of the reaction and related rate constants, which are manifested in *f_k_*(*t*), and (ii) the molecular structures of reaction intermediates, which are manifested in SADS_*k*_(*q*) that generate ΔS(*q*, *t*)_theory_ in the best agreement with ΔS(*q*, *t*)_exp_.

### B. Previous data analysis methods

In general, two types of information are desired to be extracted: (i) kinetics and (ii) molecular structures. The kinetic information, which includes the number of participating species and their associated rate constants, can also be obtained from time-resolved optical or vibrational spectroscopies. Once the kinetic information is obtained, the next step is to extract the structural information hidden in the TRXL data. The conventional analysis protocols for TRXL data can be classified into three methods: (i) linear combination fitting (LCF) with iterative least squares minimization (or iterative weighted least squares minimization), (ii) non-orthogonal decomposition (NOD), which is LCF with the direct method, and (iii) global fitting analysis (GFA).

LCF^52^ is one of the most straightforward approaches. In LCF, the difference curve at each time delay is fitted separately with a theoretical curve calculated using Eq. (9). Although *f_k_*(*t*)s are linked to ΔT(*t*) and Δρ(*t*) via the energy conservation and hydrodynamics equation,^43^ their relationships are ignored. Instead, *f_k_*(*t*), ΔT(*t*), and Δρ(*t*) are treated as independent fitting parameters. In the fitting, the chi-square (χ^2^) value representing the discrepancy between the experimental and calculated data is minimized via optimizing the fitting parameters, which are simply the proportionality coefficients of the contributing terms. Plotting the obtained proportionality coefficients of the solute terms, which correspond to *f_k_*(*t*)s, as a function of time displays the kinetic profiles of the solute molecules, and plotting those of the solvent heating terms, which correspond to ΔT(*t*) and Δρ(*t*), as a function of time delays displays how the temperature and density of the solvent changes with time, i.e., the hydrodynamic response of the solvent. This method requires prior knowledge on the identities of the solute molecules and their molecular structures because it is required to calculate SADS_*k*_(*q*)'s based on the identities and structures for the fitting. If the solute species are not known, all plausible candidate species should be tested.^49^

In the LCF method, the least squares refinement is performed for the difference curve at each time delay. Because the difference curve is a linear combination of various contributing terms, one can use linear algebra to directly determine the proportionality coefficients (direct method).^53,54^ In this way, the iterative least squares refinement can be bypassed. To distinguish this method from the LCF with the iterative minimization, we name the LCF with the direct method as “NOD.”^47^ In both LCF and NOD, the structural parameters of the candidate solute species can be varied, and in this way, the structure can also be refined. In terms of time and accuracy, NOD is advantageous over LCF with the least squares minimization, which always has a risk of being trapped in local minima.

In both LCF and NOD, the difference curve at each time delay is treated separately, with no constraints between the difference curves at different time delays. In reality, there are at least two constraints. First of all, the concentrations, *f_k_*(*t*), are not independent of one another. Instead, they should obey the kinetic rate equations of the reactions. Second, the total energy of the system should be conserved. The sum of the electronic and vibrational energy of various solute species, the energy released through radiative emissions, and the thermal energy dissipated to the bulk solvent should be conserved throughout the progress of the photoreaction via energy conservation. In GFA,^9,10,20,30,43^ these constraints can be taken advantage of by fitting the difference curves at all time delays simultaneously. Naturally, the fitting parameters are not the proportionality coefficients, *f_k_*(*t*), ΔT(*t*), and Δρ(*t*), at individual time delays, but the rate constants and some parameters required to calculate the change in the temperature and density of the solvent (such as the relative energy values of each chemical species participating in the reaction, thermodynamic parameters of the solvent, and the quantum yield of the reaction). In the fitting, the sum of the χ^2^ values at all time delays is minimized. Consequently, the total number of fitting parameters is greatly reduced. Nevertheless, this method requires prior knowledge of the solute species because SADS_*k*_(*q*)s are needed for the fitting, as in LCF and NOD. Moreover, a kinetic model needs to be assumed, and many such candidate kinetic models need to be tested via trial and error. The kinetic profiles from preliminary LCF or NOD analyses can provide a starting point for a plausible kinetic model.

### C. Drawback of previous data analysis methods

In all three methods discussed in Sec. II B, the theoretical scattering curves of solute molecules, which are used to calculate SADS_*k*_(*q*)s, need to be known or at least be assumed. In some cases, the first guess of the plausible reaction pathways and the DFT structures of the corresponding reaction intermediates work properly. Nevertheless, as the unknowns and the complexity of the studied molecular system increase, a more systematic approach is required. The simplest and most straightforward way is to test all plausible scenarios of kinetic frameworks and structures. Such an approach often works suitably, but a more efficient approach is desirable.

At this point, it should be noted that the data analysis protocol for TRXL data from proteins is quite different from that for small molecules. For the protein data, the kinetics can be first determined using a method to extract the kinetic framework, even without knowing the structures of the protein intermediates, by treating the TRXL data as if they are time-resolved spectroscopic data.^15,16,27,38,48^ Then, the resulting species-associated difference scattering curves (SADSs) consistent with the determined kinetics are subject to the subsequent structural analysis. The kinetics of proteins can be more complicated than those of small molecules. For example, the K30D mutant of homodimeric hemoglobin (HbI) exhibits one of the most complicated kinetic frameworks studied so far with TRXL.^48^ Nevertheless, the kinetics can be determined owing to various methods developed for this purpose, such as singular value decomposition (SVD) with reduced time ranges (SVD-RTR)^48^ and SVD-aided pseudo-principal-component analysis (SAPPA).^45^ The reason why the kinetics can be readily determined without any prior knowledge or guess of the molecular structures of the solute species is as follows. In the case of the protein TRXL data, the solvent term can be easily subtracted from the experimental data to yield the solute-related term because the solvent term appears in a high-*q* region where the solute-related term does not show significant contribution in the high-*q* region.^15,16,48^ A ramification from this consideration of the protein TRXL data is that even for the small-molecule data, if the solvent term can be removed, the kinetics can be readily determined from the resulting solute-related data. Toward this goal, we reported the SVD-aided non-orthogonal decomposition (SANOD) method,^47^ where the components from singular value decomposition (SVD)^55–57^ are used as the terms to be used in NOD instead of the calculated solute terms, circumventing the requirement to have prior knowledge of the solute species and their structures. The fact that the solvent term is known greatly facilitates SANOD. Nevertheless, to use the SANOD method, all necessary components need to be assembled, making its application not straightforward.

### D. A new method: Projection to extract the perpendicular component (PEPC)

**FIG. 1.**
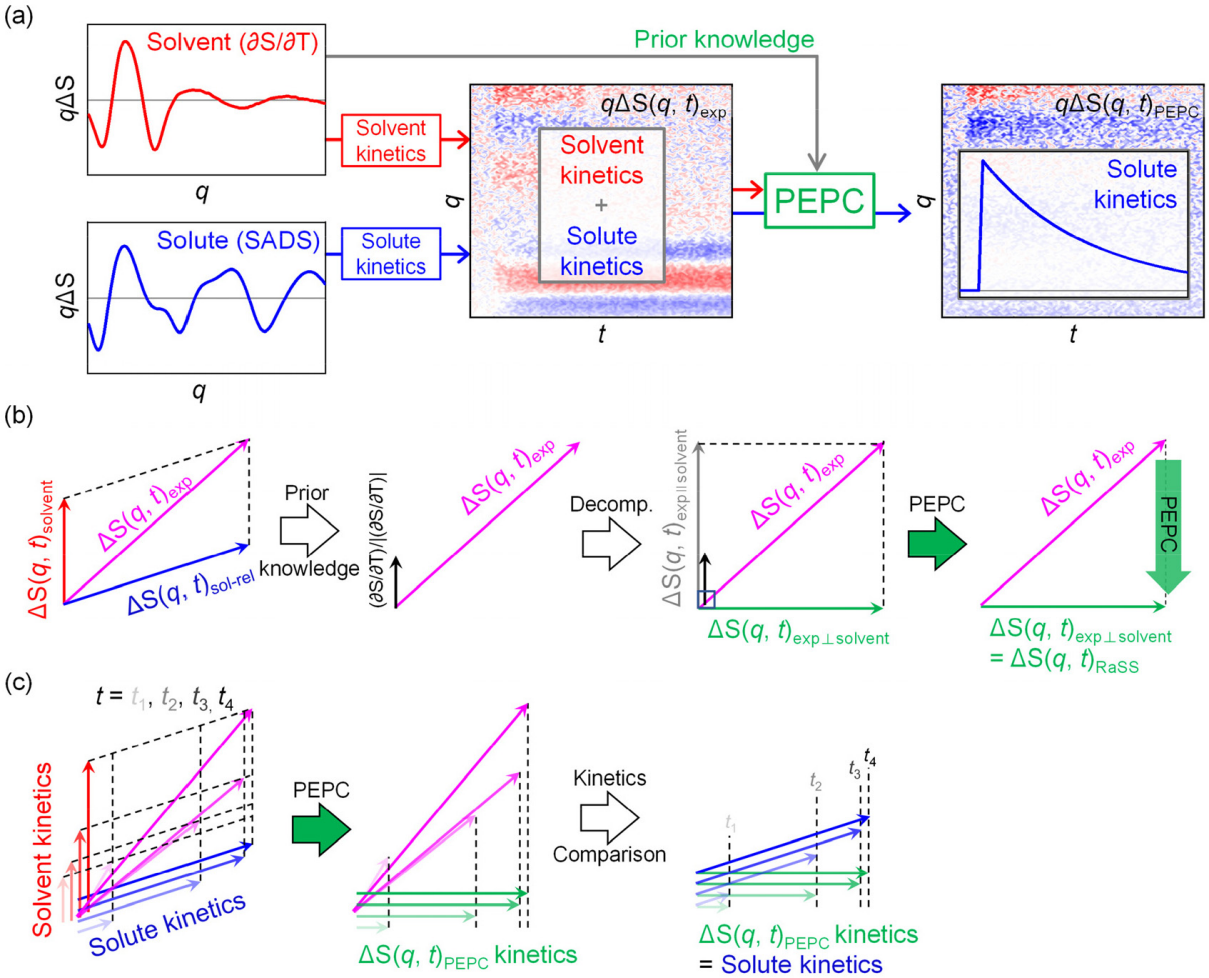
(a) Concept of the PEPC (projection to extract the perpendicular component) method. TRXL data obtained from an experiment [ΔS(*q*, *t*)_exp_, middle] are generally composed of multiple components. As a simple example, here, we assumed that ΔS(*q*, *t*)_exp_ comprises a combination of the solvent-derived (∂S/∂T)_ρ_ term (left, red) and the species-associated difference scattering curve (SADS, left, blue) arising from a single transient solute species. Each component contributes to ΔS(*q*, *t*)_exp_ with their own kinetics, making the analysis of ΔS(*q*, *t*)_exp_ complicated. The PEPC method facilitates the kinetic analysis of TRXL data by extracting the solute kinetics from ΔS(*q*, *t*)_exp_. This is achieved by removing the kinetic contribution of (∂S/∂T)_ρ_ using prior knowledge on the shape of (∂S/∂T)_ρ_ in *q*-space. (b) The signal obtained from the TRXL experiment and its components are conceptually considered as vectors and expressed by arrows. A signal obtained from the TRXL experiment [ΔS(*q*, *t*)_exp_, magenta] is composed of two different components: the solvent term [ΔS(*q*, *t*)_solvent_, red] and the solute-related term [solute term plus solute–solvent cage term, ΔS(*q*, *t*)_sol-rel_, blue]. In general, the shape of the solvent term in *q*-space (the direction of the vector) is well-known, and, therefore, the unit vector in this direction [(∂S/∂T)_ρ_/|(∂S/∂T)_ρ_|, black] is also well-known. The PEPC method removes the signal components that are parallel to the solvent term [ΔS(*q*, *t*)_exp ∥ solvent_, gray] from the total signal, leaving only the component perpendicular to the solvent term [ΔS(*q*, *t*)_exp ⊥ solvent_, green], ΔS(*q*, *t*)_PEPC_. (c) Illustration demonstrating that when the PEPC method is applied to a series of difference scattering curves measured at multiple time delays, the kinetics of the solvent term, that is, the increase or decrease in the amplitude of the solvent term with time, no longer contributes to the series of PEPC-treated difference scattering curves. Whereas the original TRXL data contain both the solute kinetics and solvent kinetics, the PEPC-treated curves contain only the solute kinetics, with no contribution from the solvent kinetics.

In the discussion so far, it has been shown that the solute kinetic model can be easily determined if the solvent term can be removed from the TRXL data. Therefore, one straightforward approach would be to remove the solvent term. However, it is generally not possible to completely remove the contribution of the solvent term from the TRXL data even if the shape of the solvent term is known. To overcome this issue, our new strategy is to remove the solvent “kinetics” from the TRXL data instead of trying to remove the solvent term itself from the TRXL data. The schematic diagram of this new strategy is illustrated in Fig. 1(a). As long as the solvent kinetics can be removed without altering the solute kinetics, the solute kinetics can be readily determined from the resulting data as demonstrated in the case of the K30D HbI,^48^ as explained in Sec. II C. In the following, we first prove that such a task not only is possible but also can be performed with a simple mathematical operation based on vector calculation.

To explain this concept, let us first consider TRXL data at a given time delay as a vector. For example, if the number of *q*-points in ΔS(*q*, *t*) is *n_q_*, then ΔS(*q*, *t*) at a single time point (*t*) can be considered as a vector in a space of *n_q_* dimensions. Since ΔS(*q*, *t*) = ΔS(*q*, *t*)_sol-rel_ + ΔS(*q*, *t*)_solvent_, both ΔS(*q*, *t*)_sol-rel_ and ΔS(*q*, *t*)_solvent_ can be also considered as vectors in the same space of *n_q_* dimensions. For the sake of simplicity, we can first consider vectors in a space of two dimensions for convenient visualization (Fig. 1). In addition, let us consider a simplistic case where ΔS(*q*, *t*)_solvent_ has only the (∂S/∂T)_ρ_ term, instead of both (∂S/∂T)_ρ_ and (∂S/∂ρ)_T_ terms, and ΔS(*q*, *t*)_sol-rel_ contains the kinetics of only a single solute species,

$${\Delta S(q,\;t)}_{\text{solvent}}=\Delta T(t)\times {\left(\partial S/\partial T\right)}_{\rho },$$ (10)

$${\Delta S(q,\;t)}_{\text{sol}–\text{rel}}=1/R\times f_{1}(t)\times {\text{SADS}}_{1}(q),$$ (11)

where ΔT(*t*) is the solvent temperature change, (∂S/∂T)_ρ_ is the solvent scattering change per unit temperature change, *f*_1_(*t*) is the molar fraction of the solute species at time *t*, and SADS_1_(*q*) is the solute-related term per unit concentration of the first solute species. In this simplistic case, the directions of ΔS(*q*, *t*)_sol-rel_ and ΔS(*q*, *t*)_solvent_ vectors do not change with time, whereas their magnitudes can change with time, making it much easier to explain the concept of our approach.

Here, a two-dimensional vector ΔS(*q*, *t*)_exp_ is the vector sum of a two-dimensional vector ΔS(*q*, *t*)_sol-rel_ and a two-dimensional vector ΔS(*q*, *t*)_solvent_, as shown in Fig. 1(b). In fact, determining ΔS(*q*, *t*)_sol-rel_ and ΔS(*q*, *t*)_solvent_ vectors is our goal, but the issue is that it is impossible to determine ΔS(*q*, *t*)_sol-rel_ and ΔS(*q*, *t*)_solvent_ vectors without prior knowledge. Here, one critical piece of prior knowledge is that we know the direction of the ΔS(*q*, *t*)_solvent_ vector, which is determined by the shape of the solvent term [(∂S/∂T)_ρ_]. In other words, the solvent unit vector [(∂S/∂T)_ρ_/|(∂S/∂T)_ρ_|], in the direction of the ΔS(*q*, *t*)_solvent_ vector, is known from (∂S/∂T)_ρ_. Therefore, we can decompose the original TRXL data [ΔS(*q*, *t*)_exp_] into components parallel [ΔS(*q*, *t*)_exp ∥ solvent_] and perpendicular [ΔS(*q*, *t*)_exp ⊥ solvent_] to the solvent unit vector using vector operations. For instance, the magnitude of the component ΔS(*q*, *t*)_exp ∥ solvent_ can be obtained by taking the dot product of ΔS(*q*, *t*)_exp_ and the solvent unit vector. Multiplying this magnitude to the solvent unit vector gives ΔS(*q*, *t*)_exp ∥ solvent_, which can be expressed as follows:

$$\begin{array}{c} {\Delta S(q,\;t)}_{\text{exp}\;∥\;\text{solvent}}=({\Delta S(q,\;t)}_{\text{exp}}\cdot \;{\left(\partial S/\partial T\right)}_{\rho }/|{\left(\partial S/\partial T\right)}_{\rho }|)\times {\left(\partial S/\partial T\right)}_{\rho }/|{\left(\partial S/\partial T\right)}_{\rho }|\\ ={\text{proj}}_{{\left(\partial S/\partial T\right)}_{\rho }/|{\left(\partial S/\partial T\right)}_{\rho }|}{\Delta S(q,\;t)}_{\text{exp}\;}={\text{proj}}_{{\left(\partial S/\partial T\right)}_{\rho }}{\Delta S(q,\;t)}_{\text{exp}}, \end{array}$$ (12)

where the symbol · denotes the dot product, and *proj*_A_B denotes the projection of a vector B to a vector A. This component parallel to the solvent unit vector [ΔS(*q*, *t*)_exp ∥ solvent_], which is the projection of the original data [ΔS(*q*, *t*)_exp_] to the solvent unit vector [(∂S/∂T)_ρ_/|(∂S/∂T)_ρ_|], is then subtracted from the original data [ΔS(*q*, *t*)_exp_] to obtain the component perpendicular to the solvent unit vector [ΔS(*q*, *t*)_exp ⊥ solvent_]. For this reason, we named this method to extract the perpendicular component as the “projection to extract the perpendicular component (PEPC).” The resulting PEPC-treated data, ΔS(*q*, *t*)_PEPC_, can be expressed as follows:

$$\begin{array}{c} {\Delta S(q,\;t)}_{\mathrm{PEPC}}={\Delta S(q,\;t)}_{\text{exp}\;⊥\;\text{solvent}}={\Delta S(q,\;t)}_{\text{exp}}-{\Delta S(q,\;t)}_{\text{exp}\;∥\;\text{solvent}}\\ ={\Delta S(q,\;t)}_{\text{exp}\;}-{\text{proj}}_{{\left(\partial S/\partial T\right)}_{\rho }}{\Delta S(q,\;t)}_{\text{exp}}. \end{array}$$ (13)

Let us now consider a series of ΔS(*q*, *t*)_exp_ vectors at multiple time delays. For typical TRXL experimental data, the magnitudes of both ΔS(*q*, *t*)_sol-rel_ and ΔS(*q*, *t*)_solvent_ change with time. Figure 1(c) shows that, even for such a case, the solvent kinetics is completely removed in ΔS(*q*, *t*)_PEPC_, and the solute kinetics, represented by the relative magnitudes of the original ΔS(*q*, *t*)_sol-rel_ vectors, is retained in the ΔS(*q*, *t*)_PEPC_ vectors.

So far, for heuristic purposes, the vectors were represented in two-dimensional space, and we considered simplistic cases where only the magnitudes of the ΔS(*q*, *t*)_solvent_ and ΔS(*q*, *t*)_sol-rel_ vectors change with time, whereas their directions do not because we intentionally supposed cases where each vector has only a single component. In real ΔS(*q*, *t*)_exp_ vectors, the *n_q_* dimensions need to be considered instead of the two dimensions. Moreover, because the ΔS(*q*, *t*)_solvent_ vectors have the (∂S/∂ρ)_T_ component as well as the (∂S/∂T)_ρ_ component, both the direction and magnitude of the ΔS(*q*, *t*)_solvent_ vectors change with time. Furthermore, because the ΔS(*q*, *t*)_sol-rel_ vectors can contain contributions from multiple solute species, their directions as well as their magnitudes will change with time. In such a general case, ΔS(*q*, *t*)_exp_ can be expressed as ΔS(*q*, *t*)_theory_ given in Eq. (9), as follows:

$${\Delta S(q,\;t)}_{\text{exp}\;}=1/R\times \sum_{k}\left(f_{k}(t)\times {\text{SADS}}_{k}(q)\right)+\Delta T(t)\times {\left(\partial S/\partial T\right)}_{\rho }+\Delta \rho (t)\times {\left(\partial S/\partial \rho \right)}_{T}.$$ (14)

Of course, at this stage, ΔT(*t*), Δρ(*t*), *f_k_*(*t*)'s, and SADS_*k*_(*q*)'s are not known. Still, conceptually, SADS_*k*_(*q*) can be decomposed into the components parallel and perpendicular to (∂S/∂T)_ρ_ and (∂S/∂ρ)_T_, as follows:

$$\begin{array}{c} {\mathrm{SADS}}_{k}(q)={\mathrm{SADS}}_{k,\;∥}(q)+{\mathrm{SADS}}_{k,\;⊥}(q)\\ =d_{k,\;T}\times {\left(\partial S/\partial T\right)}_{\rho }+d_{k,\;\rho }\times {\left(\partial S/\partial \rho \right)}_{T}+{\text{SADS}}_{k,\;⊥}(q). \end{array}$$ (15)

Here, SADS_*k*__, ∥_(*q*) represents the projection of SADS_*k*_(*q*) onto a plane containing two vectors (∂S/∂T)_ρ_ and (∂S/∂ρ)_T_, and the coefficients *d_k,_*_T_ and *d_k,_*_ρ_ correspond to the contributions of (∂S/∂T)_ρ_ and (∂S/∂ρ)_T_, respectively, to SADS_*k*__, ∥_(*q*). SADS_*k*__, ⊥_(*q*) denotes the component of SADS_*k*_(*q*) perpendicular to both (∂S/∂T)_ρ_ and (∂S/∂ρ)_T_. A detailed description of the procedure and the mathematical background for decomposing SADS_*k*_(*q*) into SADS_*k*__, ∥_(*q*) and SADS_*k*__, ⊥_(*q*) as well as the process for determining the coefficients *d_k,_*_T_ and *d_k,_*_ρ_ are provided in section “Mathematical background for the decomposition of SADS_*k*_ into SADS_*k,*__∥_ and SADS_*k,*__⊥_” of the supplementary material. Substituting Eq. (15) into Eq. (14) gives the following equation:

$${\Delta S(q,\;t)}_{\text{exp}\;}=1/R\times \sum_{k}\left(f_{k}(t)\times {\text{SADS}}_{k,\;⊥}(q)\right)+\left(\Delta T(t)+1/R\times \sum_{k}\left(f_{k}(t)\times d_{k,\;T}\right)\right)\times {\left(\partial S/\partial T\right)}_{\rho }+\left(\Delta \rho (t)+1/R\times \sum_{k}\left(f_{k}(t)\times d_{k,\;\rho }\right)\right)\times {\left(\partial S/\partial \rho \right)}_{T}.$$ (16)

Via PEPC, the vector component parallel to (∂S/∂T)_ρ_ and (∂S/∂ρ)_T_, which is $\left(\Delta T(t)+1/R\times \sum_{k}\left(f_{k}(t)\times d_{k,\;T}\right)\right)\times {\left(\partial S/\partial T\right)}_{\rho }+\left(\Delta \rho (t)+1/R\times \sum_{k}\left(f_{k}(t)\times d_{k,\;\rho }\right)\right)\times {\left(\partial S/\partial \rho \right)}_{T}$, is subtracted from the ΔS(*q*, *t*)_exp_ vector, yielding the vector component perpendicular to (∂S/∂T)_ρ_ and (∂S/∂ρ)_T_, as follows:

$$\begin{array}{c} {\Delta S(q,\;t)}_{\mathrm{PEPC}}=1/R\times \sum_{k}\left(f_{k}(t)\times {\mathrm{SADS}}_{k,\;⊥}(q)\right)\\ =1/R\times \sum_{k}\left(f_{k}(t)\times ({\text{SADS}}_{k}(q)-d_{k,\;T}\times {\left(\partial S/\partial T\right)}_{\rho }-d_{k,\;\rho }\times {\left(\partial S/\partial \rho \right)}_{T})\right). \end{array}$$ (17)

In ΔS(*q*, *t*)_PEPC_, the amounts of the subtracted solvent term are $\Delta T(t)+1/R\times \sum_{k}\left(f_{k}(t)\times d_{k,\;T}\right)$ and $\Delta \rho (t)+1/R\times \sum_{k}\left(f_{k}(t)\times \;d_{k,\;\rho }\right)$ for (∂S/∂T)_ρ_ and (∂S/∂ρ)_T_, respectively. These amounts deviate from the correct ones, ΔT(*t*) and Δρ(*t*), by $1/R\times \sum_{k}\left(f_{k}(t)\times \;d_{k,\;T}\right)$ and $1/R\times \sum_{k}\left(f_{k}(t)\times d_{k,\;\rho }\right)$, respectively. In other words, the solvent term is subtracted more than necessary, making the shapes of ΔS(*q*, *t*)_PEPC_ in the *q*-space altered from the true vector, $1/R\times \sum_{k}\left(f_{k}(t)\times {\text{SADS}}_{k}(q)\right)$. Nevertheless, as can be seen in Eq. (17), the terms contributing to the solvent kinetics, such as ΔT(*t*) and Δρ(*t*), are completely removed, and only the terms contributing to the solute kinetics, *f*_k_(*t*), are left in ΔS(*q*, *t*)_PEPC_. Therefore, a kinetic analysis such as the one applied to the TRXL data for HbI K30D^48^ can be used to extract kinetics from ΔS(*q*, *t*)_PEPC_ without any prior knowledge of the solute species and their molecular structures.

### E. Extracting the solute kinetics from ΔS(*q*, *t*)_PEPC_

Once the ΔS(*q*, *t*)_PEPC_ data are obtained via PEPC, the subsequent kinetic analysis is straightforward. Here, we provide a brief description. The first step is to use SVD on ΔS(*q*, *t*)_PEPC_ to decompose ΔS(*q*, *t*)_PEPC_ into the time-independent components in the *q*-space (left singular vectors, LSVs) and their temporal profiles in the time axis (right singular vectors, RSVs). If the experimental data have *n_q_ q*-points at each time delay and *n_t_* time delays, the SVD of these data gives a total of *n_t_* LSVs and a total of *n_t_* RSVs. However, in general, most of these LSVs and RSVs express only the noise of the experimental data, and the number of components expressing a meaningful shape on the experimental data is limited to only a few. For this reason, we first use the low-rank approximation to determine the number of meaningful components and discard the remaining noise from the experimental data prior to the kinetic analysis. The relative contributions of the decomposed vectors are represented by their singular values. Based on these singular values and the autocorrelation values, the number of significant components is determined.

The significant RSVs that survive the low-rank approximation are then fitted simultaneously with a sum of exponential functions sharing common time constants. The number of kinetic components required to satisfactorily fit RSVs and the number of significant singular vectors from the low-rank approximation play important roles as constraints in the subsequent kinetic analysis to determine the kinetic frameworks. A good example can be found in recent work on the K30D mutant of HbI.

### F. Application of PEPC to real data

**FIG. 2.**
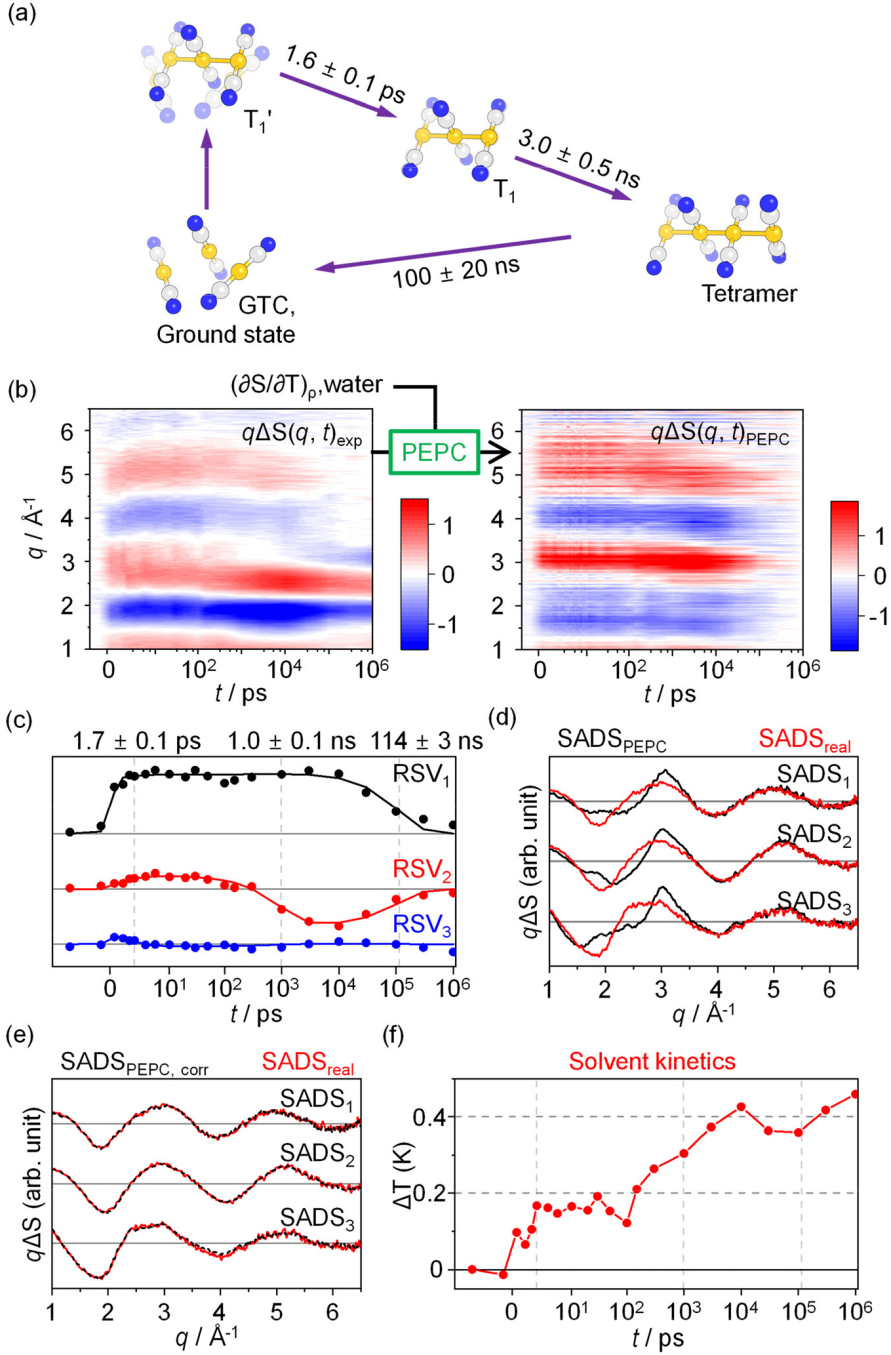
(a) Photoreaction mechanism of [Au(CN)_2_^−^]_3_ (gold trimer complex, GTC) in water, adapted from previous work.^30^ The molecular structures of the reactants and intermediates involved in the photoreaction are shown, along with the time constants and their errors. (b) ΔS(*q*, *t*)_exp_ from a TRXL experiment on [Au(CN)_2_^−^]_3_ in water,^30^ and ΔS(*q*, *t*)_PEPC_ obtained by applying the PEPC method to ΔS(*q*, *t*)_exp_. The kinetic contribution of (∂S/∂T)_ρ_ of water was eliminated using PEPC. (c) The three right singular vectors (RSVs) obtained from the SVD analysis of ΔS(*q*, *t*)_PEPC_. The dots represent the RSVs from experimental data. The three RSVs were globally fitted using a sum of three exponential functions convoluted with a Gaussian instrumental response function (IRF), yielding time constants of 1.7 ps, 1.0 ns, and 114 ns. The lines represent the theoretical fitting curves. (d) The three SADSs obtained from the kinetic analysis of ΔS(*q*, *t*)_PEPC_ (SADS_PEPC_, black solid lines) are compared with those from the global fitting analysis (GFA) of ΔS(*q*, *t*)_exp_ (SADS_real_, red solid lines). (e) Three corrected SADSs obtained by correcting the amount of (∂S/∂T)_ρ_ excessively removed in SADS_PEPC_'s (SADS_PEPC, corr_, black dash lines) are compared with those from the GFA of ΔS(*q*, *t*)_exp_ (SADS_real_, red solid lines). Each SADS_PEPC, corr_ is obtained by optimizing the weight, α, of (∂S/∂T)_ρ_ for each SADS_PEPC_ so that to minimize the discrepancy between the sum of SADS_PEPC_ and α × (∂S/∂T)_ρ_ and its corresponding SADS_real_. (f) Time-dependent changes of solvent temperature obtained by fitting ΔS(*q*, *t*)_exp_ for each *t* with the sum of the contributions of three SADS_PEPC, corr_ and ΔT(*t*) × (∂S/∂T)_ρ_, where ΔT(*t*) is the fitting parameter for each *t.*

**FIG. 3.**
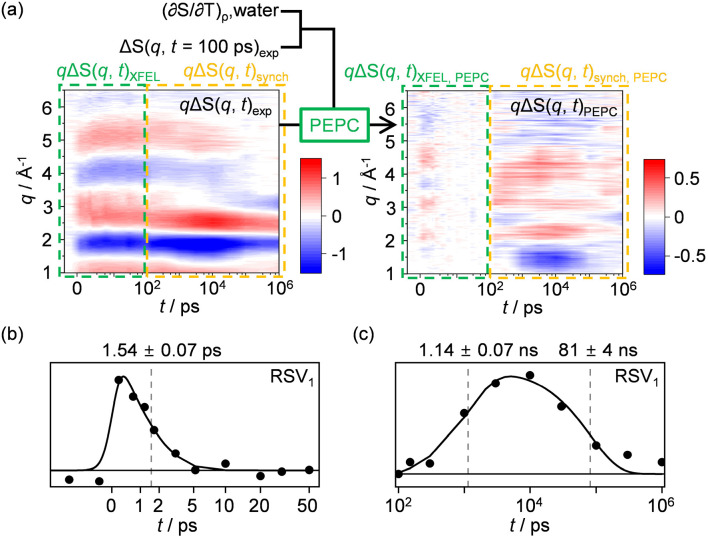
(a) ΔS(*q*, *t*)_exp_ from a TRXL experiment on [Au(CN)_2_^−^]_3_ in water,^30^ and ΔS(*q*, *t*)_PEPC_ obtained by applying the PEPC method to ΔS(*q*, *t*)_exp_. The contour plot shown for ΔS(*q*, *t*)_exp_ is the same as that shown in Fig. 2(b). Here, the kinetic contributions of two components, (∂S/∂T)_ρ_ of water and ΔS(*q*, *t* = 100 ps)_exp_, were eliminated using PEPC, leaving only the signal component perpendicular to the two components. The PEPC method is applied to two subsets of ΔS(*q*, *t*)_exp_, each corresponding to *t* < 100 ps [ΔS(*q*, *t*)_XFEL_] and *t* ≥ 100 ps [ΔS(*q*, *t*)_synch_], yielding ΔS(*q*, *t*)_XFEL, PEPC_ and ΔS(*q*, *t*)_synch, PEPC_, respectively. (b) and (c) The first RSVs obtained from the SVD analysis of (b) ΔS(*q*, *t*)_XFEL, PEPC_ and (c) ΔS(*q*, *t*)_synch, PEPC_. For (b), the first RSV was fitted using an exponential decay function with zero amplitude at *t* < 0, convoluted with a Gaussian IRF, yielding a time constant for the decay of 1.54 ps. For (c), the first RSV was fitted by the sum of an exponential rise function and an exponential decay function with time constants of 1.14 and 81 ns, respectively.

**FIG. 4.**
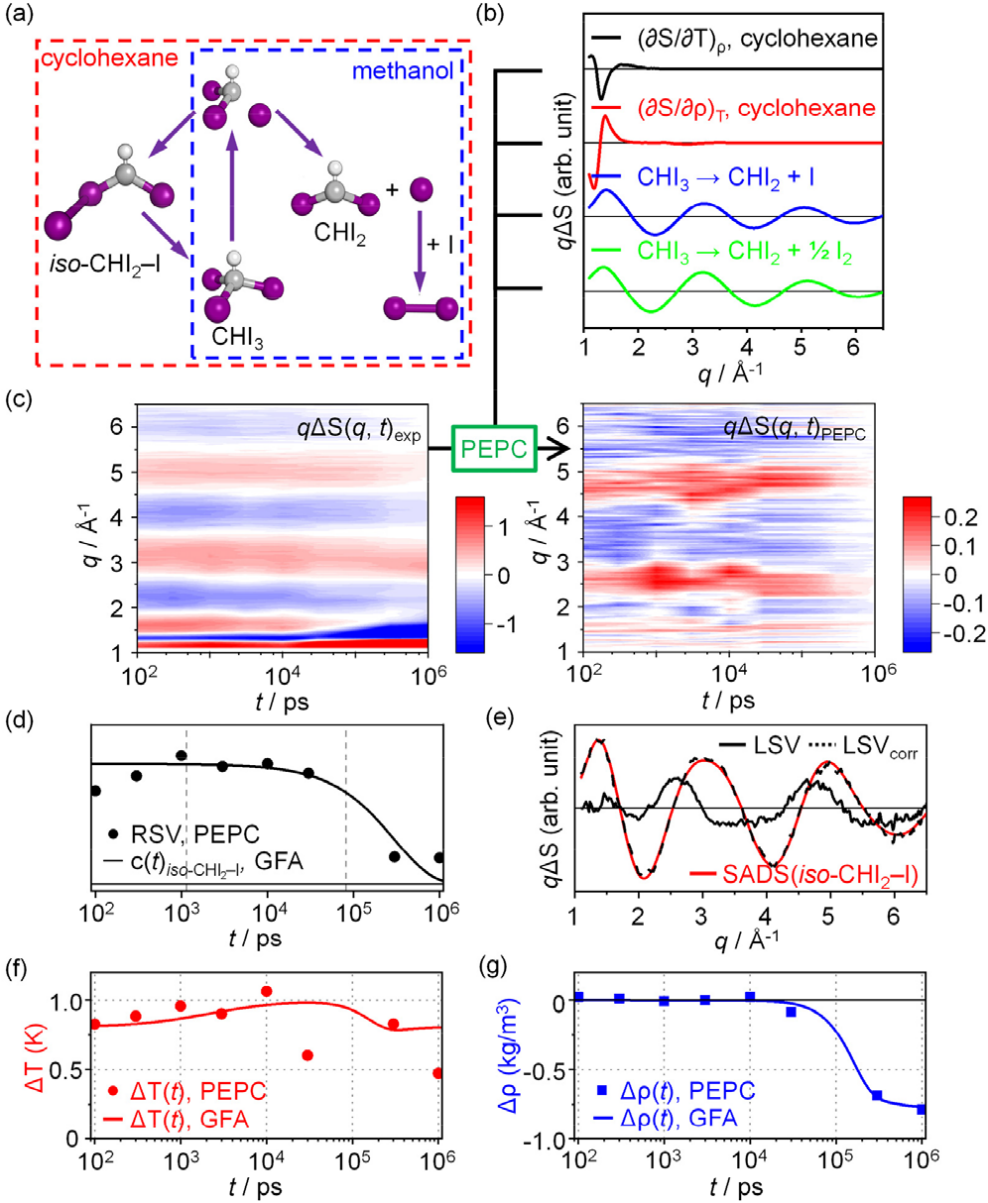
(a) Reaction pathways of CHI_3_ photolysis in methanol and cyclohexane, adapted from the literature.^9,20^ (b) Potential signal components for CHI_3_ photolysis in cyclohexane, predicted based on the null hypothesis that the reaction pathway for cyclohexane is the same as that assigned for methanol. (c) ΔS(*q*, *t*)_exp_ from a TRXL experiment on CHI_3_ in cyclohexane,^20^ and ΔS(*q*, *t*)_PEPC_ obtained by applying the PEPC method to ΔS(*q*, *t*)_exp_. The contributions of the four signal components shown in (b) are removed from ΔS(*q*, *t*)_exp_ via PEPC. The remaining signal in ΔS(*q*, *t*)_PEPC_ indicates that the null hypothesis is not true, and the reaction pathways are different for cyclohexane and methanol. (d) The first RSV obtained from the SVD analysis of ΔS(*q*, *t*)_PEPC_ (dots). The kinetics of the first RSV agrees well with the time-dependent concentration profile of *iso*-CHI_2_–I retrieved using GFA (solid line), indicating that the remaining signal in ΔS(*q*, *t*)_PEPC_ is due to the contribution of the reaction pathway yielding *iso*-CHI_2_–I. (e) The first LSV obtained from the SVD analysis of ΔS(*q*, *t*)_PEPC_ (black solid line). The LSV is compared with the SADS corresponding to the formation of *iso*-CHI_2_–I [SADS(*iso*-CHI_2_–I), red solid line]. Although the first LSV did not correspond well with SADS(*iso*-CHI_2_–I), the LSV_corr_ (black dashed line), which was adjusted for the contributions of the signal components excessively removed during the PEPC procedure, showed excellent agreement with SADS(*iso*-CHI_2_–I). The LSV_corr_ is described as the sum of five terms, LSV, α × (∂S/∂T)_ρ_, β × (∂S/∂ρ)_T_, γ × SADS(CHI_3_ → CHI_2_ + I), and δ × SADS(CHI_3_ → CHI_2_ + ½ I_2_) where α, β, γ, and δ are the fitting parameters. (f) and (g) The time traces of ΔT(f) and Δρ(g), retrieved from the correct kinetic framework that includes the contribution of *iso*-CHI_2_–I (dots). Once the kinetics of the reaction is determined using PEPC and the solute-related term for each relevant chemical species is determined through a subsequent structural analysis, the reconstruction of the hydrodynamic response of the solvent is straightforward. ΔT and Δρ for each time delay can be retrieved as described in the text. The resulting ΔT and Δρ match those determined using GFA (solid lines).

To demonstrate the effectiveness of the PEPC method, we applied the technique to actual experimental data obtained from the photoreaction of the gold trimer complex (GTC), [Au(CN)_2_^−^]_3_ in water.^30^ The results of GFA of the data have been reported.^30^ The photoreaction pathway revealed from the GFA, including the molecular structure of the reactants and intermediates, as well as the time constants of the reaction pathways, is shown in Fig. 2(a). We employed the PEPC technique to process the original ΔS(*q*, *t*)_exp_, which led to the generation of PEPC-treated ΔS(*q*, *t*)_PEPC_ data as depicted in Fig. 2(b). For PEPC, the signal component which is parallel to (∂S/∂T)_ρ_ of water is removed from ΔS(*q*, *t*)_exp_. Normally, the solvent term consists of two signal components (∂S/∂T)_ρ_ and (∂S/∂ρ)_T_, and, thus, the signal component which is parallel to either of the two signal components should be removed in the PEPC method. Nevertheless, here, only one of the two components (∂S/∂ρ)_T_ is considered and removed in the PEPC method because (∂S/∂T)_ρ_ and (∂S/∂ρ)_T_ for water have similar shapes.^30^ The shape of (∂S/∂T)_ρ_ is obtained from a separate experiment using a dye solution (FeCl_3_ dissolved in water). It should be noted that, in Fig. 2 and in all subsequent figures, the difference scattering curves, ΔS(*q*, *t*), are displayed in the form of *q*ΔS(*q*, *t*), i.e., ΔS(*q*, *t*) multiplied by the magnitude of the momentum-transfer vector, *q*, to better visualize the small signals at large *q* values. SVD analysis was performed to investigate the solute-only kinetics in ΔS(*q*, *t*)_PEPC_. Examination of the singular values, autocorrelation values, LSVs, and RSVs indicates that the first three components make a substantial contribution to ΔS(*q*, *t*)_PEPC_ (Fig. S1). The three major RSVs obtained from the SVD analysis of ΔS(*q*, *t*)_PEPC_ are shown in Fig. 2(c). A satisfactory fit of the RSVs of ΔS(*q*, *t*)_PEPC_ gives three time constants of 1.7 ± 0.1 ps, 1.0 ± 0.1 ns, and 114 ± 3 ns. The time constants are in excellent agreement with those identified from the previous study using GFA (1.6 ± 0.1 ps, 3.0 ± 0.5 ns, and 100 ± 20 ns)^30^ except for some discrepancy in the second time constant. The three components and three time constants immediately suggest a kinetic model with three species in sequential transitions. Hence, we used this kinetic model and applied kinetics-constrained analysis (KCA) to obtain the SADSs of the three species. We note that when KCA is applied to extract SADSs, a specific kinetic model is applied, and SADSs compatible with the applied kinetic model are extracted. The extracted SADSs are not equal to LSVs. Instead, each SADS must be a linear combination of LSVs. The resulting SADSs (SADS_PEPC_) are shown in Fig. 2(d) (black solid lines). When compared to the SADSs for the three intermediates identified from the previous GFA [SADS_real_, Fig. 2(d), red solid lines],^30^ at first glance, the shape of each SADS_PEPC_ is noticeably different from its corresponding SADS_real_. Nevertheless, the determined kinetic framework and the time constants are consistent with the reported ones obtained from the GFA. The excellent agreement shows that the solute kinetics can be easily obtained from ΔS(*q*, *t*)_PEPC_. One might notice that the contour plot of the PEPC-treated data in Fig. 2(b) [and Figs. 3(a) and 4(c) to be presented later] exhibits some horizontal stripes. We note that these stripes are not generated by the PEPC procedure but rather are present in the original data, ΔS(*q*, *t*)_exp_, except for the case of Fig. 3(a). The stripes are less visible in ΔS(*q*, *t*)_exp_ due to its higher overall signal amplitude compared to that of ΔS(*q*, *t*)_PEPC_. In the case of Fig. 3(a), the horizontal stripes were significantly enhanced with the PEPC procedure. However, the increase in the amplitude of the artifact features is not inherent to the PEPC method itself but is a result of the poor signal-to-noise ratio of the signal components used in PEPC, particularly ΔS(*q*, *t* = 100 ps)_exp_. A more in-depth discussion of this issue is presented in the “Horizontal stripes in the contour plot of the PEPC-treated data” section of the supplementary material.

### G. Structural analysis using the SADSs from ΔS(*q*, *t*)_PEPC_

When the kinetic model is determined in this way, SADSs corresponding to each species suitable for the model can be obtained. By applying structural analysis to these SADSs, information on the structure of each species can be obtained. It should be noted that the PEPC process preserves the solute kinetics but alters the shapes of SADSs in *q*-space, as mentioned in Sec. II D. Therefore, the alteration of SADSs needs to be taken into account in the structural analysis based on the SADSs obtained from ΔS(*q*, *t*)_PEPC_, denoted as SADS_*k*_(*q*)_PEPC_. The alteration of the shapes of SADS_*k*_(*q*)_PEPC_ is caused because the solvent term is excessively removed in the PEPC process, as given in Eq. (17). Here, SADS_*k*_(*q*)_PEPC_ and SADS_*k*_(*q*)_real_ correspond to SADS_*k*__, ⊥_(*q*) and SADS_*k*_(*q*) in Eq. (17), respectively.

This relation is expressed in the following equation:

$$\begin{array}{c} {\mathrm{SADS}}_{k}{\left(q\right)}_{\mathrm{PEPC}}={\mathrm{SADS}}_{k,\;⊥}(q)={\mathrm{SADS}}_{k}(q)-d_{k,\;T}\times {\left(\partial S/\partial T\right)}_{\rho }-d_{k,\;\rho }\times {\left(\partial S/\partial \rho \right)}_{T}\\ ={\text{SADS}}_{k}{\left(q\right)}_{\text{real}}-d_{k,\;T}\times {\left(\partial S/\partial T\right)}_{\rho }-d_{k,\;\rho }\times {\left(\partial S/\partial \rho \right)}_{T}. \end{array}$$ (18)

This equation shows that in SADS_*k*_(*q*)_PEPC_, the (∂S/∂T)_ρ_ term is further removed by *d_k_*_, T_ × (∂S/∂T)_ρ_, and the (∂S/∂ρ)_T_ term is further removed by *d_k_*_, ρ_ × (∂S/∂ρ)_T_. Considering this, it is necessary to conduct structural analysis using the amount of solvent term excessively removed in the PEPC process as a fitting parameter. The corrected SADSs can be expressed as follows:

$${\text{SADS}}_{k}{\left(q\right)}_{\text{corr}}={\text{SADS}}_{k}{\left(q\right)}_{\text{PEPC}}+g_{k}\times {\left(\partial S/\partial T\right)}_{\rho }+h_{k}\times {\left(\partial S/\partial \rho \right)}_{T}.$$ (19)

Here, *g_k_* and *h_k_* are the coefficients for (∂S/∂T)_ρ_ and (∂S/∂ρ)_T_, respectively, and ideally should be the same as *d_k_*_, T_ and *d_k_*_, ρ_, respectively. Then, in the fitting process, the χ^2^ value representing the discrepancy between SADS_*k*_(*q*)_corr_ and SADS_*k*_(*q*) is minimized using *g_k_* and *h_k_* as well as the structural parameters of intermediates and reactants which are used to calculate S_*k*_(*q*)_solute_, S_0_(*q*)_solute_, S_*k*_(*q*)_cage_, and S_0_(*q*)_cage_ in Eq. (7), as fitting parameters. By correcting the contribution of the solvent term that is excessively removed in the PEPC process, specifically *g_k_* × (∂S/∂T)_ρ_ and *h_k_* × (∂S/∂ρ)_T_, the shape of the difference scattering curve for each species obtained from the GFA technique^30^ can be reproduced [Fig. 2(f)]. Here, the contribution of *h_k_* × (∂S/∂ρ)_T_ is ignored for this specific example with water as a solvent. It is noteworthy that, despite adjusting *g_k_* and *h_k_* to minimize the discrepancy between SADS_*k*_(*q*)_corr_ and SADS_*k*_(*q*), the satisfactory fitting of SADS_*k*_(*q*)_corr_ to SADS_*k*_(*q*) is only achieved for the correct structure of the species, thus enabling the identification of the species and optimization of the detailed molecular structure. For instance, Fig. S5 highlights that SADS_1_(*q*)_corr_ is only consistent with SADS calculated for the structure corresponding to the T_1_′ state and exhibits a noticeable deviation from those calculated for the structures corresponding to the T_1_ state or the tetramer (Fig. S5, left panels). Similarly, SADS_2_(*q*)_corr_ and SADS_3_(*q*)_corr_ match only to SADS calculated for the structures corresponding to the T_1_ state and the tetramer, respectively (Fig. S5, middle and right panels). These findings unequivocally demonstrate that SADS_*k*_(*q*)_corr_ accurately matches SADS calculated only for the correct molecular structure of the species. In other words, despite the altered shape of SADS_*k*_(*q*)_PEPC_ in *q*-space, it remains feasible to extract the relevant structural information corresponding to each species by analyzing SADS_*k*_(*q*)_PEPC_.

With this, both the kinetic analysis and the structural analysis for the solute term are completed. Here, we note that consideration of *g_k_* and *h_k_* in the structural fitting using PEPC-treated data does not increase the complexity of the structural fitting. In other words, *g_k_* and *h_k_* are not independent fitting parameters in the structural fitting but are functions expressed by independent fitting parameters. Accordingly, the structural analysis of a SADS_*k*_(*q*)_PEPC_ has the same level of complexity (the number of independent fitting parameters) as the structural analysis of a correct SADS, a SADS_*k*_(*q*), that is not altered by the PEPC process. The method for determining the coefficients *g_k_* and *h_k_* is described in section “Determination of coefficients *g_k_* and *h_k_* during structural analysis” of the supplementary material.

The last remaining step is to reconstruct the information about the hydrodynamic response of the solvent (solvent kinetics). The analysis of solvent kinetics is straightforward once the solute-related term, ΔS(*q*, *t*)_sol-rel_, is determined from the kinetic analysis and structural analysis. At this stage, *f_k_*(*t*) for the solute-related term in Eq. (5) is obtained through kinetic analysis. The subsequent structural analysis refines the molecular structures and determines SADS_*k*_(*q*). The solvent-only term, ΔS(*q*, *t*)_solvent_ = ΔT(*t*) × (∂S/∂T)_ρ_ + Δρ(*t*) × (∂S/∂ρ)_T_, can be determined by subtracting ΔS(*q*, *t*)_sol-rel_, i.e., 1/*R* × Σ_k_[*f_k_*(*t*) × SADS_*k*_(*q*)], from ΔS(*q*, *t*)_exp_, as shown in Eq. (14). ΔT(*t*) and Δρ(*t*) can be retrieved from the ΔS(*q*, *t*)_solvent_ by simply performing NOD of ΔS(*q*, *t*)_solvent_ as a linear combination of two terms (∂S/∂T)_ρ_ and (∂S/∂ρ)_T_. In the case of the [Au(CN)_2_^−^]_3_ in water shown earlier, the profile of ΔT(*t*) reconstructed through the corresponding process is shown in Fig. 2(f). Note that the two terms (∂S/∂T)_ρ_ and (∂S/∂ρ)_T_ for the solvent response have similar shapes in *q*-space, especially in the case of water, making it difficult to distinguish the contributions of the two terms in ΔS(*q*, *t*)_solvent_. Accordingly, here, the hydrodynamics response is described with only the (∂S/∂T)_ρ_ term, as in Eq. (10), instead of the linear combination of the two terms, and the resulting ΔT(*t*) is indicated. In general, except for water, the two terms (∂S/∂T)_ρ_ and (∂S/∂ρ)_T_ for the solvent response have distinct shapes in *q*-space, and, therefore, the contribution of each term in the solvent response can be easily determined. An example of such a general case, CHI_3_ in cyclohexane, is given in Figs. 4(f) and 4(g).

### H. PEPC for simultaneous removal of the contributions of multiple signal components

As demonstrated in Sec. II F, PEPC is a powerful tool for eliminating the contribution of a single component, such as (∂S/∂T)_ρ_. In fact, PEPC can also be used to simultaneously remove multiple signal components, making it a versatile method with applications extending beyond solvent subtraction. For example, PEPC can be employed to eliminate the contributions of known, trivial reaction intermediates, allowing for the isolation of potential contributions from unknown intermediates that may be hidden in experimental signals. Additionally, PEPC can be applied to eliminate the kinetic contributions of an intermediate dominating at a specific time delay, enabling the extraction of contributions from other intermediates in the reaction.

Prior to discussing applications of PEPC, we provide an overview of its theoretical principles. We describe how PEPC can eliminate the contributions of multiple signal components, which we refer to as “trivial components” for brevity, and provide a mathematical representation of the resulting PEPC-treated signal. The vector space spanned by these trivial components is referred to as the “trivial space (TS).” Assuming the presence of *m* trivial components, denoted as {*trv*_1_, *trv*_2_, …, *trv_m_*}, PEPC eliminates signal components parallel to these *m* trivial components and retains only the component perpendicular to all *m* components. The relation between ΔS(*q*, *t*)_exp_ and ΔS(*q*, *t*)_PEPC_ can be expressed as follows:

$${\Delta S(q,\;t)}_{\text{exp}\;}=\sum_{i=1}^{m}w_{i}\times {\text{trv}}_{i}+{\Delta S(q,\;t)}_{\text{PEPC}}.$$ (20)

Here, *w_i_* represents the weight of *i*th trivial component, *trv_i_*, removed via PEPC. As illustrated in Fig. 1(c), the kinetics of a trivial component does not contribute in the direction perpendicular to that component. Therefore, by finding ΔS(*q*, *t*)_PEPC_ that is perpendicular to all *m* trivial components, the kinetics of the signal components other than the *m* trivial components can be extracted. This can be achieved by satisfying the following equation for all *i*:

$${\Delta S(q,\;t)}_{\text{PEPC}}\;\cdot {\;\text{trv}}_{i}=0.$$ (21)

The main concern is whether a set {*w*_1_, *w*_2_, …, *w_n_*} for Eq. (20) that satisfies Eq. (21) for all *i* can be readily calculated.

In fact, the set {*w*_1_, *w*_2_, …, *w_n_*} that satisfies Eq. (21) for all *i* can be readily obtained by calculating the mathematical projection. The projection of a vector *v* onto a subspace U, spanned by a set of vectors {*u*_1_, *u*_2_, …, *u_n_*}, denoted by *proj*_U_*v*, provides the vector component that lies within the subspace, while the residual of the projection, i.e., *v* − *proj*_U_*v*, gives the component perpendicular to all vectors, *u*_1_, *u*_2_, …, and *u_n_*.^58^ Thus, the first term on the right-hand side of Eq. (20) can be obtained by calculating the projection, *proj*_TS_ΔS(*q*, *t*)_exp_, and the second term, ΔS(*q*, *t*)_PEPC_, that satisfies Eq. (21) can be obtained by calculating the residual of the projection, ΔS(*q*, *t*)_exp_ − *proj*_TS_ΔS(*q*, *t*)_exp_. Mathematically, this can be expressed as follows:

$${\Delta S(q,\;t)}_{\text{PEPC}}={\Delta S(q,\;t)}_{\text{exp}\;}-\sum_{i=1}^{m}w_{i}\times {\text{trv}}_{i}={\Delta S(q,\;t)}_{\text{exp}\;}-{\text{proj}}_{\mathrm{TS}}{\Delta S(q,\;t)}_{\text{exp}}.$$ (22)

Equation (22) shows how PEPC can eliminate the contributions of multiple trivial components, retaining only the component perpendicular to the trivial components by removing signal components parallel to the trivial components. Mathematically, this PEPC treatment can be expressed using a projection operator. However, the projection formula may not be very intuitive in practice. To address this, we propose a practical and intuitive method based on a mathematical property of the projection operator: the projection, *proj*_U_*v*, is given by the linear combination of {*u*_1_, *u*_2_, …, *u_n_*} that minimizes the magnitude of the residual, *v* − *proj*_U_*v*.^58^ By utilizing this property, we can obtain the projection, *proj*_TS_ΔS(*q*, *t*)_exp_, by finding the linear combination of {*trv*_1_, *trv*_2_, …, *trv_m_*} that minimizes the magnitude of the residual of the projection, ΔS(*q*, *t*)_PEPC_. Based on this, the projection, *proj*_TS_ΔS(*q*, *t*)_exp_, can be obtained by finding the least squares solution of the following system of linear equations:^59–61^

$${\Delta S(q,\;t)}_{\text{exp}\;}=\sum_{i=1}^{m}x_{i}\times {\text{trv}}_{i}.$$ (23)

Here, *x_i_* represents the weight of *i*th trivial component, *trv_i_*. Note that the least squares solution, which we denote as {*w*_LS, 1_, *w*_LS, 2_, …, *w*_LS,__*m*_}, can be obtained unambiguously based on well-established numerical algorithms.^62^ Once the least squares solution is determined, ΔS(*q*, *t*)_PEPC_ can be derived as follows:

$${\text{proj}}_{\mathrm{TS}}{\Delta S(q,\;t)}_{\text{exp}\;}=\sum_{i=1}^{m}w_{LS,\;i}\times {\text{trv}}_{i},$$ (24)

$${\Delta S(q,\;t)}_{\text{PEPC}}={\Delta S(q,\;t)}_{\text{exp}\;}-{\text{proj}}_{\mathrm{TS}}{\Delta S(q,\;t)}_{\text{exp}\;}={\Delta S(q,\;t)}_{\text{exp}\;}-\sum_{i=1}^{m}w_{LS,\;i}\times {\text{trv}}_{i}.$$ (25)

This approach based on least squares fitting provides a straightforward and practical method for numerically calculating the PEPC-treated signal, ΔS(*q*, *t*)_PEPC_, for multiple trivial components, {*trv*_1_, *trv*_2_, …, *trv_m_*}.

The kinetic analysis of ΔS(*q*, *t*)_PEPC_ resulting from the PEPC treatment with multiple trivial components can be performed in the same way as for ΔS(*q*, *t*)_PEPC_ treated with a single trivial component, namely, (∂S/∂T)_ρ_. Once the kinetic framework is established from the kinetic analysis, the SADS corresponding to the kinetic framework can be obtained using KCA. To extract molecular structural information, the resulting SADS_*k*_(*q*)_PEPC_ can be analyzed similarly, with corrections similar to those in Eq. (19) but with the equation modified as follows:

$${\text{SADS}}_{k}{\left(q\right)}_{\text{corr}}={\text{SADS}}_{k}{\left(q\right)}_{\text{PEPC}}+\sum_{i=1}^{m}\alpha_{i,\;k}\times {\text{trv}}_{i}.$$ (26)

Here, α_*i*_, _*k*_ is the weight of the correction for the *i*th trivial component, *trv_i_*. By employing Eq. (26), the molecular structure of each species can be optimized by minimizing the deviation between the experimentally obtained SADS_*k*_(*q*)_corr_, which has been corrected for the distortion from PEPC treatment, and the calculated SADS_*k*_(*q*)_real_ derived from the molecular structure.

### I. Application of PEPC to determine the kinetic framework

To showcase the versatility of PEPC, particularly in handling multiple trivial components, we utilized it in analyzing two distinct datasets: one for the photoreaction of [Au(CN)_2_^−^]_3_ in water^30^ and the other for the photoreaction of CHI_3_ in cyclohexane.^20^ For [Au(CN)_2_^−^]_3_ in water, the TRXL data consist of two sub-datasets measured at two different facilities: a synchrotron and an XFEL.^30^ The data corresponding to *t* ≥ 100 ps [ΔS(*q*, *t*)_synch_] and *t* < 100 ps [ΔS(*q*, *t*)_XFEL_] were obtained from the experiments at the synchrotron and XFEL, respectively. Typically, a synchrotron experiment using the same sample is performed before the XFEL experiment. In this case, the preliminary information, ΔS(*q*, *t*)_synch_, can be used when analyzing ΔS(*q*, *t*)_XFEL_ to identify any contributions from short-lived intermediates that were not identified in ΔS(*q*, *t*)_synch_. Moreover, when analyzing ΔS(*q*, *t*)_synch_, PEPC can be used to identify any contributions from long-lived intermediates other than those present at the earliest time delay, *t* = 100 ps, by removing the contribution of the intermediate present at *t* = 100 ps from the entire ΔS(*q*, *t*)_synch_. Here, in contrast to the PEPC process illustrated in Fig. 2(b), the contribution of ΔS(*q*, *t* = 100 ps)_exp_ is removed, in addition to the contribution of solvent heating signal (∂S/∂T)_ρ_. As a result, only the signal component perpendicular to these two components remains after removing the signal components parallel to (∂S/∂T)_ρ_ and ΔS(*q*, *t* = 100 ps)_exp_. The resulting ΔS(*q*, *t*)_PEPC_ from ΔS(*q*, *t*)_XFEL_ and ΔS(*q*, *t*)_synch_ is shown in Fig. 3(a) as ΔS(*q*, *t*)_XFEL, PEPC_ and ΔS(*q*, *t*)_synch, PEPC_, respectively. The observation of a remaining signal in ΔS(*q*, *t*)_XFEL, PEPC_ and ΔS(*q*, *t*)_synch, PEPC_ suggests the presence of reaction intermediates other than those exists at *t* = 100 ps, which was previously assigned as an intermediate in the T_1_ state based on GFA.^30,34^ SVD analysis revealed that each of ΔS(*q*, *t*)_XFEL, PEPC_ and ΔS(*q*, *t*)_synch, PEPC_ exhibits only one significant LSV (Figs. S6 and S7), indicating the presence of an additional early intermediate before the intermediate in the T_1_ state within the *t* < 100 ps range and the other late intermediate in the *t* > 100 ps range. The significant RSVs are shown in Figs. 3(b) and 3(c). Kinetic analysis of the RSV of ΔS(*q*, *t*)_XFEL, PEPC_ reveals that the RSV rises instantaneously within the instrument response function (IRF, 480 fs) of the experiment and decays with a single time constant of 1.54 ± 0.07 ps. Obviously, this observation indicates that the early intermediate (an intermediate in the T_1_′ state)^34^ forms within the IRF and transforms to the intermediate in the T_1_ state with a 1.54 ps time constant. For ΔS(*q*, *t*)_synch, PEPC_, the RSV rises with a 1.14 ± 0.07 ns time constant and decays with an 81 ± 4 ns time constant, indicating that the T_1_ intermediate transforms to the other late intermediate (a tetramer)^30^ with 1.14 ns, and the late intermediate decays to ground state with 81 ns.

From the description of the kinetic analysis earlier, one would notice that the kinetic analysis of two sub-datasets, ΔS(*q*, *t*)_XFEL, PEPC_ and ΔS(*q*, *t*)_synch, PEPC_, is even more straightforward than that of ΔS(*q*, *t*)_PEPC_ demonstrated in Sec. II F. This is due to two reasons. First, the data are divided into two subsets and analyzed separately, thereby reducing the numbers of signal components and time constants in each sub-dataset. Specifically, ΔS(*q*, *t*)_XFEL, PEPC_ has one signal component and one time constant, and ΔS(*q*, *t*)_synch, PEPC_ has one signal component and two time constants, compared to the three signal components and three time constants in the entire ΔS(*q*, *t*)_PEPC_ shown in Fig. 2(b). However, the advantage of dividing the data into subsets for kinetic analysis will not be further discussed in this study, as it is not relevant to the benefit of using PEPC. Second, and more importantly, PEPC eliminates the contribution of an additional trivial component, i.e., ΔS(*q*, *t* = 100 ps)_exp_, reducing the number of signal components, i.e., the number of relevant chemical species contributing to the signal. Thus, applying PEPC reduces the number of solute signal components in ΔS(*q*, *t*)_XFEL_ and ΔS(*q*, *t*)_synch_ from two [T_1_′ and T_1_ in ΔS(*q*, *t*)_XFEL_ and T_1_ and tetramer in ΔS(*q*, *t*)_synch_] to one [T_1_′ only in ΔS(*q*, *t*)_XFEL_ and tetramer only in ΔS(*q*, *t*)_synch_]. Consequently, the kinetics of the remaining solute species, T_1_′ and tetramer, is more clearly exposed in the resulting significant RSVs of ΔS(*q*, *t*)_XFEL, PEPC_ and ΔS(*q*, *t*)_synch, PEPC_, respectively, facilitating the kinetic analysis.

The photoreaction of CHI_3_ presents a fundamental question concerning whether the reaction pathway depends on the solvent, and if so, how. Therefore, it is crucial to determine whether the reaction pathways differ in different solvents. Previous studies examined the reaction pathway in methanol using TRXL [Fig. 4(a), pathways wrapped in a blue dashed line], identifying that only the dissociation channel yielding the CHI_2_ radical and I radical or the iodine molecule is active.^9^ Afterward, the same reaction was examined in a different solvent, cyclohexane, using TRXL.^20^ In the study, the reaction pathway in cyclohexane was identified by using GFA, confirming that the pathway is different in cyclohexane [Fig. 4(a), pathways wrapped in a red dashed line].

If one were to analyze the TRXL data for cyclohexane, again, an essential question to address is whether the reaction pathway differs from that observed in methanol. To address this question using GFA, one would need to verify whether the fitting using the kinetic framework determined for methanol works well with the TRXL data for cyclohexane, which requires optimizing several parameters such as rate constants and excitation ratio. However, such a verification task can be tedious and time-consuming. In this study, we demonstrate that PEPC offers a more straightforward approach for confirming any differences in reaction pathways between solvents.

The key is that, by applying PEPC, the contributions of the signal components corresponding to the reaction pathways identified for methanol can be removed from the TRXL data for cyclohexane, allowing for an assessment of whether the reaction pathway differs in the two solvents. Under the null hypothesis that the reaction pathway for cyclohexane is the same as that assigned for methanol, it can be predicted that the two solute-related terms corresponding to the formation of CHI_2_ and the iodine radical [Fig. 4(b), blue solid line] and the subsequent transformation of the iodine radical to the iodine molecule [Fig. 4(b), green solid line] as well as the two solvent terms of cyclohexane [Fig. 4(b), black and red solid lines for (∂S/∂T)_ρ_ and (∂S/∂ρ)_T_ of cyclohexane, respectively] would contribute to the TRXL signal for the photolysis of CHI_3_ in cyclohexane. PEPC enables removal of the contributions of these four signal components from ΔS(*q*, *t*)_exp_, and the resulting ΔS(*q*, *t*)_PEPC_ can be used to test the null hypothesis. If the null hypothesis is true, then there would be no meaningful signal in the ΔS(*q*, *t*)_PEPC_. However, if the reaction pathway is different and a signal component not identified for methanol exists, then a significant amplitude of the signal would remain in the ΔS(*q*, *t*)_PEPC_, indicating the presence of a distinct signal component that was not be completely subtracted during the PEPC process.

The contour plots of the measured ΔS(*q*, *t*)_exp_ for cyclohexane and the ΔS(*q*, *t*)_PEPC_ are depicted in Fig. 4(c). The presence of a remaining signal in ΔS(*q*, *t*)_PEPC_ suggests that the null hypothesis is invalid, and the reaction pathways differ for cyclohexane and methanol. Further investigation, involving kinetic and structural analysis of the residual signal in ΔS(*q*, *t*)_PEPC_, reveals that the residual signal originates from the pathway that produces an isomer of CHI_3_, *iso*-CHI_2_–I,^20^ a pathway that is inactive in methanol^9^ [Fig. 4(a)]. SVD analysis of ΔS(*q*, *t*)_PEPC_ results in a single major component (Fig. S8). Regarding kinetics, the major RSV obtained from the SVD analysis [Fig. 4(d), dots] matches with the time-dependent concentration profile of *iso*-CHI_2_–I, obtained using GFA^20^ [Fig. 4(d), solid line], indicating that the residual signal in ΔS(*q*, *t*)_PEPC_ is due to *iso*-CHI_2_–I. Furthermore, regarding structural analysis, the LSV_corr_ [Fig. 4(e), black dashed line] calculated based on Eq. (26), i.e., LSV_corr_ = LSV + α × (∂S/∂T)_ρ_ + β × (∂S/∂ρ)_T_ + γ × SADS(CHI_3_→CHI_2_ + I) + δ × SADS(CHI_3_→CHI_2_ + ½ I_2_), where α, β, γ, and δ are the fitting parameters, agrees excellently with the SADS calculated for *iso*-CHI_2_–I^20^ [SADS(*iso*-CHI_2_–I), Fig. 4(e), red solid line], thus confirming that the residual signal in ΔS(*q*, *t*)_PEPC_ originates from *iso*-CHI_2_–I.

The demonstration of PEPC to the TRXL data for the photoreaction of CHI_3_ shows how PEPC can be utilized to determine the kinetic framework of the reaction and to analyze the structural dynamics of the reaction. The significant residual signal observed in ΔS(*q*, *t*)_PEPC_ confirms the difference in the reaction pathways for cyclohexane and methanol. Further analysis of ΔS(*q*, *t*)_PEPC_ reveals (1) a reaction intermediate, *iso*-CHI_2_–I, that is involved in the reaction pathways in cyclohexane, along with (2) the kinetics of *iso*-CHI_2_–I, especially, when and how *iso*-CHI_2_–I forms and decays, and (3) the molecular structure of *iso*-CHI_2_–I. The determined kinetics and molecular structures can be utilized for the analysis of the hydrodynamics of the solvent, cyclohexane, as explained in Sec. II G. The resulting ΔT(*t*) and Δρ(*t*) are shown in Figs. 4(f) and 4(g), respectively.

Although we have presented and demonstrated the PEPC method as a powerful tool for analyzing the kinetic behavior of TRXL signals, it is worth noting that a conventional method, referred to as the “nodal-point method (NP method),” can also provide similar benefits. However, as discussed in the “Comparison of the PEPC method and the nodal-point method (NP method)” section of the supplementary material, our comparison shows that the PEPC method outperforms the NP method in several crucial aspects.

### J. Potential of the PEPC method for other types of data

In this study, we demonstrated the ability of the PEPC method to remove the solvent kinetics from the TRXL data by utilizing the prior knowledge of the shape of the solvent term. In principle, the application of PEPC is not limited to TRXL data and can be extended to other types of data where a certain component is known. By using the PEPC method, the kinetics of the known component can be removed regardless of the type of the data. Furthermore, the PEPC method can be applied to datasets other than time-resolved data. In time-resolved data, spectra change with time. For other types of data, the spectra change with experimental variables other than time, such as temperature or pressure. In such cases, if the spectrum of a species is known and its magnitude changes with the experimental variable, the PEPC method can be used to subtract its variable-dependent changes from the original data. For example, for a set of spectra measured as a function of temperature, the temperature-dependent changes in the spectrum of a species can be removed using the PEPC method. Overall, the PEPC method has a great potential as a versatile and powerful tool for the analysis of various types of data, extending beyond the scope of TRXL data analysis.

## III. CONCLUSION

In this work, we introduced PEPC, a new analytic method for the analysis of time-resolved data, and demonstrated its application through various examples. By removing the kinetic contributions of known signal components, such as solvent heating or well-known intermediates or products, the PEPC technique greatly facilitates the kinetic analysis of the treated signal. As a representative example, we showed that the PEPC technique can be used to easily extract kinetic information of solute-related terms from TRXL data by removing the kinetic contributions of solvent terms. Although the solvent term being excessively removed in this process alters the shape of the solute-related term in *q*-space, structural analysis can still be performed on the resulting PEPC-treated data to obtain structural information. As the PEPC process is fully arithmetic, it has a fast computation time, allowing for real-time monitoring of the status of the data being obtained during the experiment. Overall, the PEPC technique has the potential to serve as a valuable tool for both post-experiment data analysis and real-time monitoring of time-resolved data, with the ability to extend beyond the scope of TRXL data analysis. Further research is required to explore and demonstrate the full range of applications of this powerful and versatile method in various fields of science.

## SUPPLEMENTARY MATERIAL

See the supplementary material for detailed discussions on various topics, including the mathematical background for the decomposition of SADS_*k*_ into SADS_*k,*__∥_ and SADS_*k,*__⊥_, the appearance of horizontal stripes in the contour plot of the PEPC-treated data, the determination of coefficients *g_k_* and *h_k_* during structural analysis, and a comparison of the PEPC method and the nodal-point method.

## Data Availability

The data that support the findings of this study are available from the corresponding author upon reasonable request.
